# Microbial Quality of Sliced Pawpaw (*Carica papaya*) and Watermelon (*Citrullus lanatus*) Sold on Some Streets of Accra Metropolis, Ghana

**DOI:** 10.1155/2021/6695957

**Published:** 2021-01-26

**Authors:** Michael Olu-Taiwo, Baakwa Miah De-Graft, Akua Obeng Forson

**Affiliations:** Department of Medical Laboratory Science, School of Biomedical and Allied Health Sciences, College of Health Sciences, University of Ghana, Korle-bu, Ghana

## Abstract

In most African countries, street vending of fruits is prevalent and the likelihood of predisposing consumers to microbial contamination is very high. This study aimed to determine various bacteria and risk factors that are associated with fruits sold by street vendors in Accra. Sliced watermelons and pawpaws were randomly purchased from selected suburbs in Greater Accra Region of Ghana. One gram (1 g) of each watermelon and pawpaw was homogenized in 9 ml of sterile peptone water, and 0.1 ml from each serial dilutions of each fruit was spread on plate count agar, blood agar, and MacConkey agar plates for total aerobic counts and coliform counts. Agar plates were incubated at 33–37°C for 18–24 h. Bacterial identification was done by standard bacteriological methods. Additionally, questionnaires were administered to the vendors to gather data on food hygiene and knowledge on foodborne illness. The study revealed that although some of the fruit vendors were educated on food hygiene, most sold fruits were contaminated with mean total aerobic plate counts of 2.6 × 10^5^–8.1 × 10^5^ CFU g^−1^ and 3.7 × 10^4^–7.1 × 10^4^ CFU g^−1^ for watermelon and pawpaw. The mean coliform counts for pawpaw and watermelon ranged between 1.2 × 10^3^–8.1 × 10^3^ CFU g^−1^ and 1.6 × 10^4^–3.1 × 10^4^ CFU g^−1^, respectively. Overall, mean aerobic counts and mean coliform counts were not significantly different among vendors in selected locations (*p* > 0.05). However, predominant bacteria isolated included *Enterobacter* species (33.3%), *Citrobacter* sp. (20.0%), and *Klebsiella* sp. (15.9%). The study revealed that watermelon and pawpaw sold on the streets in Accra could be possible source of foodborne illness. Therefore, street food vendors must be educated on food hygiene protocols and measures to improve microbial quality of street vended fruits.

## 1. Introduction

In the last few years, there has been a substantial increment in consumption of sliced/ready-to-eat fruits in Ghana, due to its affordability in small quantity, convenience, freshness, easy accessibility, and nutritional benefits [1–3]. The high consumption pattern of sliced fruits has led to an escalation of foodborne illness [4]. Sliced fruits particularly consumed in Accra include pawpaw, pineapple, watermelon, apple, and mango. Street vendors often slice fresh fruits by peeling and cutting them into pieces and packing them in small polythene bags to be sold to consumers for immediate consumption without necessarily rinsing or washing them because they have already been packaged. Most of the street vendors have a low educational level, unlicensed and untrained in food hygiene [2]. Despite the benefits derived from daily consumption of fruits, they are prone to microbial contaminations in addition to the associated risk of disease transmission to which consumers may be exposed. Furthermore, various pathogenic bacterial species have been associated with contaminations of fruits and vegetables as evidenced by their isolation from several fruits and vegetables [1, 5, 6]. According to CDC [7], watermelon caused *Salmonella* outbreak in 2002 and 2006 in the United States. Likewise, in 2011, watermelon from Brazil was implicated in outbreak of *Salmonella* infections in Europe, with 63 confirmed cases of food poisoning [8]. In Nigeria, a study to determine the microbiological quality of sliced watermelon sold by street vendors revealed an average aerobic counts of 0.1–2.3 × 10^5^ CFU g^−1^ [3]. Another study in Nigeria has isolated *Staphylococcus aureus*, *Klebsiella* sp., *Salmonella* sp., and *Escherichia coli* in ranges between 9.0 × 10^5^ and 3.0 × 10^7^ CFU/ml from carrot, runner bean, cucumber, fresh cut pineapple, green pepper, cabbage, spring onions, lettuce, watermelon, and apple [6]. Furthermore, Pesewu et al. [9] study on raw-mixed vegetable salads in the Accra Metropolis reported a high level of bacterial contamination with *Escherichia coli* (36%), *Staphylococcus aureus* (33%), *Klebsiella* sp. (17%), and *Bacillus* sp. (15%). However, despite all the awareness of microbial contamination of fruits and vegetables, there is paucity of information in Ghana on potential risk factors and microbial quality of street vended sliced polyethylene packaged fruits. This study aimed to determine the bacterial quality of polyethylene packed sliced pawpaw and watermelon sold by street vendors in Accra and to assess risk factors associated with their consumption.

## 2. Materials and Methods

### 2.1. Study Participant

Packaged sliced watermelon and pawpaw were randomly purchased and sampled from March to April, 2017. Street fruit vendors were selected at Korle-Bu, University of Ghana (night market), Agbogbloshie, Shiashi, and Accra Tema Station, all in the Greater Accra Region. Only prewashed/sliced watermelon and pawpaw packaged in polyethylene bags or transparent microwave bowls by vendor were collected. A total of 45 sliced watermelons and 45 sliced pawpaws were used for this study. Nine samples each of pawpaw and watermelon were taken from each location. The samples were transported on ice packs within two hours to the University of Ghana, School of Biomedical and Allied Health Sciences Microbiology Laboratory. In addition, questionnaires were administered to vendors to gather data on vendor's personal hygiene, food hygiene, knowledge on foodborne illness and their causes.

### 2.2. Bacteriological Analysis of Samples

Approximately, 1 g of fresh cut fruits (pawpaw and watermelon) was sliced with a sterile knife and added to 9 ml of 0.1% buffered peptone water to prepare homogenate suspension using stomacher bag (Seward, UK). This was followed by a ten-fold serial dilution of 10^−2^ to 10^−4^ of homogenized samples as described by Nwachukwu and Osuocha [2]. An aliquot of 0.1 ml from each serial dilutions was inoculated onto plate count agar (Oxoid, Cambridge, UK), blood agar (Oxoid, Cambridge, UK), and MacConkey agar (Oxoid, Cambridge, UK) using the spread plate technique for total viable aerobic count and coliform count. Inoculation was done in duplicates, and the plates were incubated at 33–37°C for 24–48 h [2].

### 2.3. Bacteria Identification

Identification of bacterial isolates was done by first subculturing from primary culture plates to obtain pure culture colonies. Bacteria identification from pure culture plates was based on colonial morphology, and a representative colony on each plate was picked and Gram stained and further tested using indole, methyl red, citrate, oxidase, Voges–Proskauer tests, urease and coagulase tests [10]. An API 20E identification system (bioMerieux SA, Marcy l‟Etoile, France) was also used to confirm all the Gram negative isolates.

### 2.4. Bacterial Colony Enumeration

Enumeration of bacterial colonies was carried out from plate count agar for total aerobic count and total coliform count from MacConkey agar. After incubation, colonies that were in the range between 30 and 300 were considered.

### 2.5. Data Analysis

Bacterial counts were calculated as colony forming units per gram (CFU g^−1^) and then converted into log10 values. Descriptive statistics were computed using the Microsoft Excel data analysis, Fischer's exact, and chi-square, and one-way ANOVA was analyzed using GraphPad Prism software, version 6, to find the statistical difference (*P* < 0.05). Statistical significance was set at a *P* value of <0.05.

## 3. Result

### 3.1. Demographics and Characteristics of Fruit Vendors

All the watermelon vendors for this study were females. However, 71.1% vendors were aged between 30 and 49 years, 24.4% vendors were below 30 years old, and 4.4% of vendors were above 50 years old (Table 1). Sixty-eight percent (31/45) of the vendors had received formal education with the commonest level of education being junior high school (31.1% (14)). Most of the watermelon vendors were mobile [42.2% (19/45)], and 26.7% (12/45) were stationary at their various vending sites (Table 1).

Ninety-seven percent of the pawpaw vendors were females, and 2.2% were males. Fifteen percent of the pawpaw fruit vendors were below the age of 30 years, 13.3% were above 50 years, and 71.1% vendors were between 30 and 49 years (Table 1). Twenty percent of the vendors had attended basic school, 15.6% had junior high school, 20.0% had senior high school, 2.2% had tertiary education, and 42.2% gave no response regarding their educational level. Most (66.7%) of the vendors were stationary, and 24.4% were mobile vendors (Table 1).

### 3.2. Risk Factors and Personal Hygiene of Fruit Vendors

Regarding personal hygiene, 15.6% of the watermelon vendors washed their hands at least 3 times a day, while 44.4% washed their hands more than 3 times in a day (Table 2). Seventy-three percent of the vendors washed their hands before and after peeling or slicing the watermelons. Also, 62.2% (28/45) vendors washed their fruits before packaging and slicing, while 11.1% did not. Sixty-eight percent of the vendors washed their knife before using them, and 71.1% washed their tray before using them. Seventy-three percent of the watermelon vendors used tap water to wash their watermelon and packaged their sliced watermelon with polyethylene bags. Only 13.3% of the vendors had attended a course on food hygiene, but 86.6% had not attended any course on food hygiene.

Thirteen percent of the pawpaw vendors washed their hands at least three times in a day, while 35.6% washed their hands more than 3 times in a day. Twenty-two percent of the pawpaw vendors claimed they had vending experience of less than 10 years, 20% had between 10 and 20 years, and 13.3% had vending experience of more than 20 years. Most (75.6%) of the pawpaw vendors washed their hands before and after peeling and slicing the pawpaws (Table 2). Sixty-six percent of the vendors washed their fruits before slicing and packaging them, and 33.3% did not wash their fruits before slicing and packaging. Also, 73.3% of the vendors washed their knives before using them and 68.9% said they washed their trays before using them. Concerning courses on food hygiene, only 8.9% had attended some courses on food hygiene at the time of the study. Six percent of the vendors kept leftover sliced fruits covered up on the table for the next day, while 17.8% of the vendors discarded any leftover fruits at the close of the day. No statistical association was found for the different assessed risk factors associated with water melon and pawpaw vendors (*P* > 0.05).

### 3.3. Bacteriological Count

#### 3.3.1. Mean Total Aerobic Count

Overall, the mean aerobic count for pawpaw and watermelon samples analyzed ranged between 3.7 × 10^4^ and 8.1 × 10^5^ CFU g^−1^. The mean aerobic count for watermelon ranged between 2.6 × 10^5^ and 8.1 × 10^5^ CFU g^−1^, while the mean aerobic count for pawpaw ranged between 3.7 × 10^4^ CFU g^−1^ and 8.3 × 10^4^ CFU g^−1^. The maximum mean aerobic count was observed in watermelon and pawpaw from Korle-Bu (8.1 × 10^5^ and 8.3 × 10^4^ CFU g^−1^). While the minimum mean aerobic count was observed in pawpaw from Accra Tema Station (3.7 × 10^4^ CFU g^−1^). Mean aerobic counts were not significantly different among vendors in selected locations (*p* > 0.05) (Table 3).

#### 3.3.2. Mean Total Coliform Count

Overall, the mean coliform count for pawpaw and watermelon ranged between 1.2 × 10 and 1.6 × 10^4^ CFU g^−1^. The maximum mean coliform count was observed in watermelon from Korle-Bu (1.6 × 10^4^ CFU g^−1^), while minimum mean coliform count was exhibited in pawpaw from Shiashi (1.2 × 10^3^ CFU g^−1^). Mean coliform counts were not significantly different among vendors in selected locations (*p* > 0.05) (Table 3).

### 3.4. Bacteria Isolated from Packaged Sliced Watermelons at Various Locations

Bacteria isolated from sliced watermelons were *Citrobacter koseri* (1.5%), *Citrobacter* sp. (19.4%), *Enterobacter* sp. (28.4%), *Klebsiella pneumoniae* (7.5%), *Klebsiella* sp. (20.9%), *Proteus vulgaris* (3.0%), *Pseudomonas* sp. (1.5%), *Staphylococcus aureus* (7.5%), and *Staphylococcus epidermidis* (10.5%) (Table 4).

Comparing isolates from the various selling locations, Korle-Bu had the highest number of isolates (45.0%) (Table 4). Bacterial isolates from Korle-Bu include *Citrobacter* sp. (7), *Enterobacter* sp. (9), *Klebsiella pneumoniae* (1), *Klebsiella* sp. (8), *Proteus vulgaris* (2), *Staphylococcus aureus* (1), and *Staphylococcus epidermidis* (2). Thirty percent 30% isolates which included *Citrobacter koseri* (1), *Citrobacter* sp. (3), *Enterobacter* sp. (6), *Klebsiella pneumoniae* (1), *Klebsiella* sp. (4), *Pseudomonas* sp. (1), *Staphylococcus aureus* (3), and *Staphylococcus epidermidis* (1) were isolated from watermelon from Agbogbloshie Market.

### 3.5. Bacteria Isolated from Sliced Pawpaws at Various Sales Locations

Bacteria isolated from pawpaws were *Citrobacter koseri* (4.6%), *Citrobacter* sp. (20.0%), *Enterobacter* sp. (38.5%), *Klebsiella pneumoniae* (6.2%), *Klebsiella* sp. (10.8%), *Pseudomonas* sp. (3.1%), *Staphylococcus epidermidis* (1.5%), and *Staphylococcus aureus* (15.4%) (Table 4).

Pawpaw sampled from Korle-Bu had the highest contamination of 31% (21) including *Citrobacter koseri* (1), *Citrobacter* sp. (4), *Enterobacter* sp. (8), *Klebsiella pneumoniae* (1), *Klebsiella* sp. (4), and *Staphylococcus aureus* (3). Also, a total of 29% (19) were obtained from University of Ghana (night market) and from Shiashi, and eight isolates (12%) were from pawpaw, whilst 17% (11) of isolates were obtained from Agbogbloshie, including *Citrobacter koseri* (2), *Citrobacter* sp. (2), *Enterobacter* sp. (4), and *Klebsiella* sp. (3). However, 9% (6) of isolates were obtained from Accra Tema Station, including *Citrobacter* sp. (2), *Enterobacter* sp. (3), and *Klebsiella pneumoniae* (1).

## 4. Discussion

In this study, 98% of street fruit vendors were females and most of them were within 30–49 years of age (71.1%) with some form of formal education but with limited knowledge on food hygiene and safety. These study findings are in agreement with Odonkor et al. [11] and Donkor et al. [12] findings that reported 76% and 98.4% of females are involved in the business of street vending of foods. In contrast to these findings, a study by Mudey et al. [13] in India, has reported 69.4% of males were engaged in ready-to-eat foods. A knowledge in food hygiene and safety and the consequences of contaminated or spoiled fruits on the health of the consumers should be a major concern to the business of fruit vendors and the state at large [14]. In Ghana, metropolitan assemblies collaborate with the Food and Drug Authority (FDA) and officials of respective municipal and district assemblies for street vendors to obtain vending authorization [15]. These authorization includes obtaining a development and building permit (DBP) from the Accra Metropolitan Assembly (AMA) and business operating permit (BOP) from health inspectors from the Environmental Health Department following an inspection of the hygiene of the place of vending and a valid personal health certificate issued by a recognized health institution [14, 15]. However, compared to other countries, there is no need to attend a hazard analysis and critical control point (HACCP) training course before authorization is given [16]. Findings from this study have revealed the need for emphasis on food safety education for street fruit vendors and hawkers in order to eradicate harm to consumers' health.

The consumption of vended foods is a risk for foodborne illness to consumers health because it is difficult to ascertain the hygienic processes the fruits were subjected to after harvesting, during processing, and before packaging [15]. This study found 62.2% and 60.7% of the vendors washed their watermelon and pawpaw, respectively, before slicing and packaging of the fruits, whilst 68.9% of the watermelon and 73.3% of the pawpaw vendors washed their knives before using them to slice the fruits. The contamination of the fruits in this study may have been introduced by the vendors using the same bucket of tap water to wash the fruits before slicing and packaging or using the same contaminated knife to cut the fruits into smaller pieces before displaying or selling them to customers [16, 17]. Polyethylene bags which were the commonly used packages for the sliced fruits are waterproof and chemically resistant [17]. However, the sliced pawpaw and watermelon were still contaminated. Bacteria found in the polyethylene packaged sliced pawpaw and watermelon could also have been introduced from exposure of sliced watermelon to contaminated environments before they were processed and packaging [18].

Most studies usually report the presence of pathogens in fruits and vegetables, but the bacterial counts are often not documented [19]. Aerobic plate count can be used to monitor the hygienic quality of products throughout processing and distribution [20]. In this study, watermelon had a higher level of bacterial contamination (2.6 × 10^5^–8.1 × 10^5^ CFU g^−1^) as compared to pawpaw (3.7 × 10^4^ CFU g^−1^–7.1 × 10^4^ CFU g^−1^). This study's findings for pawpaw are comparable to that of Abisso et al. [21] study in South Ethiopia which reported a mean aerobic count of 4.2 × 10^2^–2.8 × 10^4^ CFU/ml in papaya fruit juice. Our findings is in contrast to a study in Nigeria which reported a higher total aerobic count of 4.2 × 10^5^–1.4 × 10^6^ CFU g^−1^ and 4.1 × 10^5^ CFU g^−1^-1.6 × 10^6^ CFU g^−1^ for pawpaw and watermelon, respectively [5]. Furthermore, a higher mean aerobic count of >6 × 10^5^ CFU g^−1^ for pawpaw and watermelon has been reported in a similar study in Oman [22]. However, another study in Nigeria had reported a lower mean aerobic count of 1.5 × 10^2^–3.6 × 10^3^ CFU g^−1^ in watermelon juice [23]. The International Commission on Microbiological Specifications for Foods (ICMSF) has proposed that ready-to-eat foods with mean aerobic counts between 0 and 10^3^ are acceptable, within 10^4^–10^5^ are tolerable, and ≥10^8^ are unacceptable [24]. In this study's findings, the level of contamination could therefore be tolerable based on the recommended standards. In this study, watermelon had higher levels of bacteria occurrence than pawpaw. This could be due to the fact that watermelon grows close to the soil and may be susceptible to microbiological contamination from soil [25]. Furthermore, coliforms were detected in all samples analyzed, with an overall mean total coliform counts ranging between 1.2 × 10^3^ and 3.1 × 10^4^ CFU g^−1^. Watermelon had higher coliform counts of 1.6 × 10^4^–3.1 × 10^4^ CFU g^−1^ as compared to pawpaw counts of 1.2 × 10^3^–8.1 × 10^3^ CFU g^−1^. The possible reason for the higher coliform counts in watermelon may be due to the fact that watermelons are cultivated close to soil and this can enable easy contact of watermelon fruit with manure and irrigation water that may be contaminated.

In this study, *Enterobacter* sp. was the predominant bacteria isolated from both sliced watermelon (28.4%) and pawpaw (38.5%). This finding is slightly higher than the 21.4% prevalence in vegetables reported by Adebayo-Tayo and Okonko, [23] in Nigeria. A study with mango and papaya juices by Abisso et al. [21] in South Ethiopia has previously reported the presence of *Enterobacter* sp. Furthermore, in a Ghanaian review of ready-to-eat foods, *Enterobacter* sp. was observed as the predominant isolated bacteria. [26]. Other bacteria isolated from pawpaw and water melon were *Citrobacter* sp., *Klebsiella* sp., *Klebsiella pneumoniae*, *Staphylococcus aureus*, and *Staphylococcus epidermidis* which conforms to a previous study conducted in Nigeria [2, 27]. The presence of these bacteria on sliced fruits can be associated with exposure of the sliced fruits to the environment and contamination during processing [28, 29]. Activities such as slicing and packaging and transporting of fruits for sales have been found to be major risk factors of bacteria contamination [16]. A study with fruits and vegetables by Al-Kharousi et al. [22] in Oman revealed 60% of fruits and 91% of vegetables were contaminated with *Enterobacteriaceae*. The least bacteria isolated from the watermelon in that study was *Pseudomonas* sp. (1.5%) and from the pawpaw was *Staphylococcus epidermidis* (1.5%). A previous study conducted by Chukwu et al. [27] in Nigeria has also reported the presence of *Pseudomonas aeruginosa*, *Klebsiella* sp., *Proteus* sp., and *Enterobacter* sp. on precut pawpaw and watermelon. *Pseudomonas* sp., *Klebsiella* sp., *Proteus* sp., and *Enterobacter* sp. are bacteria commonly associated with plants, human skin, animals, and dairy products [30]. The presence of these bacteria in sliced watermelon and pawpaw may have been introduced by the contaminated hands of the vendors or by the use of contaminated water during washing and processing of the precut fruits [31, 32]. The presence of *Staphylococcus aureus* in watermelon and pawpaw is in conformity with studies from Nigeria [2, 21]. *Staphylococcus epidermidis* and *Staphylococcus aureus* are normal flora of the body [33]. The presence of *Staphylococcus epidermidis* and *Staphylococcus aureus* in the sliced fruits could be as a result of contamination from hands used for the processing and packaging of the sliced fruit [27, 33]. *Staphylococcus aureus* is known to cause food poisoning and disease presentation is mostly characterized by nausea, vomiting, and diarrhoea [34]. In addition, the consumption of contaminated food harbouring staphylococcal endotoxins [34], produced when *Staphylococcus aureus* loads exceed 10^5^ CFU g^−1^ can also be very fetal [35]. While buying the fruits from the vendors, it was observed that the knife used in cutting the watermelon before packaging was not washed but kept in a polyethylene bag and repeatedly used throughout the day without intermittent washing. This practice might have introduced the bacteria onto the sliced pawpaw and watermelon. Generally, watermelon and pawpaw can be contaminated when the utensils used for processing the fruits are not cleaned appropriately [33].

## 5. Conclusion

Polyethylene packaged sliced watermelon and pawpaw sold on the streets in some selected sites in Accra were found to be contaminated with bacteria. Contamination of the fruits may have resulted from poor handling of the fruits during preparation. Therefore, street food vendors must be educated on food hygiene protocols and measures to improve microbial quality of street vended fruits. This is because safety of ready-to-eat foods and fruits is absolutely mandatory since it contributes to economic prosperity, boosts tourism and sustainable developments. Hence, food safety should be a major priority to all governments over the world.

## 6. Strength and Weaknesses

This study reports different microbial organisms are associated with some vended fruits (watermelon and pawpaw) in Ghana. Finding are vital in creating awareness on possible risks associated with the consumption of ready to eat packaged fruits. To a greater extent, this study has provided information to consummers and will intern assist in reducing the outbreak of diseases and help spark future studies that will aid in control of microbial contamination in street vended fruits in Africa.

The study focused on the total aerobic count, total coliform count, and identification of isolated bacteria types associated with polyethylene packed sliced pawpaw and watermelon sold by street vendors in Accra. The total aerobic count of bacteria was of marginal acceptable levels and could pose threat to the health of consumers. Limited samples from few vending sites in the Regional Capital of Ghana were tested thereby not allowing extrapolation of our results to other regions and to help determine the sources of contamination. The actual harm of the contaminated fruits to the consumers' health was not proven since they were not followed up to ascertain the effects of the bacteria contamination on their health.

## Figures and Tables

**Table 1:** Demographic characteristics of the fruit vendors.

Parameters	Site	Watermelon	Pawpaw
		No. (%)	No. (%)
*Selling point of fruit*	KB	21 (46.7)	12 (26.7)
	AB	13 (28.9)	8 (17.8)
	UNM	7 (15.6)	13 (28.9)
	ATS	2 (4.4)	6 (13.3)
	SH	2 (4.4)	3 (6.7)
	TM	0	3 (6.7)

*Sex*			
	Male	0	1 (2.2)
	Female	45 (100)	44 (97.8)

*Age*			
	<30	11 (24.4)	7 (15.6)
	30–49	32 (71.1)	32 (71.1)
	≥50	2 (4.4)	6 (13.3)

*Educational status*			
	Basic school	11 (24.4)	9 (20)
	JHS	14 (31.1)	7 (15.6)
	SHS	5 (11.1)	9 (20.0)
	Tertiary	1 (2.2)	1 (2.2)
	No answer	14 (31.1)	19 (42.2)

*Type of vendor*			
	Stationary	12 (26.7)	30 (66.7)
	Mobile	19 (42.2)	11 (24.4)
	No answer	14 (31.1)	4 (8.9)

*Vending years' experience*			
	5–10	21 (46.7)	10 (22.2)
	11–20	8 (17.7)	9 (20.0)
	>20	1 (2.2)	6 (13.3)

ATS: Accra Team Station; AB: Agbogbloshie; UNM: University of Ghana Night Market; SH: Shiashi; KB: Korle-Bu; TM: Tuesday Market.

**Table 2:** Fruit vendors of packaged pawpaw and watermelon and associated risk factors.

Risk factors		Watermelon, *n* (%)	Pawpaw, *n* (%)
*Hand washing*	At least three times daily	7 (15.6)	6 (13.3)
	More than three times daily	20 (44.4)	16 (35.6)
	Wash hands before peeling fruits	Yes = 33 (73.3), no = 12 (26.7)	Yes = 34 (75.6), No = 11 (24.4)
	Wash hands after peeling fruits	Yes = 33 (73.3), no = 12 (26.7)	Yes = 34 (75.6), No = 11 (24.4)

*Handling procedures*			
	Wash fruit before slicing and packaging	Yes = 28 (62.2), no = 16 (35.6)	Yes = 30 (60.7), no = 15 (33.3)
	Wash knife before using	Yes = 31 (68.9), no = 13 (28.9)	Yes = 33 (73.3), no = 12 (26.7)
	Wash display tray before usage	Yes = 32 (71.1), no = 14 (31.1)	Yes = 31 (68.9), no = 14 (31.1)

*Water for handling*			
	Tap water	33 (73.3)	34 (75.6)
	No answer	12 (26.7)	11 (24.4)

*Type of package used*	Polyethylene bag	33 (73.3)	34 (75.6)
	No answer	12 (26.7)	11 (24.4)

*Methods of storage*			
	All fruits are sold out	27 (60.0)	34 (75.6)
	Discarded	6 (13.3)	3 (6.7)
	Covered up in a room	12 (26.7)	8 (17.8)

*Courses on food hygiene*			
	Yes	6 (13.3)	4 (8.9)
	No	25 (55.6)	21 (46.7)
	No answer	14 (31.1)	20 (44.4)

*How long ago the course was attended*			
	<1 year	5 (11.1)	4 (8.9)
	>2 years	1 (2.2)	0
	No response	39 (86.7)	41 (91.1)

**Table 3:** Mean aerobic counts and mean coliform counts of packaged pawpaw and watermelon from various locations.

Location	N	Mean	Mean	Mean	Mean	N	Mean	Mean	Mean	Mean
		Ac CFU g^−1^		Ac CFU g^−1^			CC CFU g^−1^		Ac CFU g^−1^	
		Pawpaw	Log 10	Watermelon	Log10		Pawpaw	Log10	Watermelon	Log 10
KB	9	7.1 × 10^4^	4.85	8.1 × 10^5^	5.90	9	8.1 × 10^3^	4.14	2.4 × 10^4^	4.38
AB	9	8.3 × 10^4^	4.91	7.3 × 10^5^	5.80	9	6.2 × 10^3^	3.87	1.5 × 10^4^	4.17
UNM	9	5.4 × 10^4^	4.73	6.3 × 10^5^	5.87	9	4.6 × 10^3^	3.70	3.1 × 10^4^	4.49
SH	9	4.3 × 10^4^	4.60	5.3 × 10^5^	5.73	9	1.2 × 10^3^	3.07	1.4 × 10^4^	4.14
ATS	9	3.7 × 10^4^	4.52	2.6 × 10^5^	5.41	9	2.4 × 10^3^	3.38	1.6 × 10^4^	4.20
Total	45					45				

^*∗*^Mean aerobic and mean coliform counts were not significantly different among vendors in selected locations (*p* > 0.05). ATS: Accra Team Station; AB: Agbogbloshie; UNM: University of Ghana Night Market; SH: Shiashi; KB: Korle-Bu; AC: aerobic count; CC: coliform count.

**Table 4:** Frequency of isolates from packaged watermelon and pawpaw at various locations.

Isolated obtained	Packaged watermelon					Packaged pawpaw					
	ATS	AB	UNM	SH	KB	ATS	Ab	UNM	SH	KB	Total (%)
*Citrobacter koseri*	0	1	0	0	0	0	2	0	0	1	4 (3.0)
*Citrobacter* sp.	1	3	2	0	7	2	2	3	2	4	26 (20.0)
*Enterobacter* sp.	1	6	1	2	9	3	4	9	1	8	44 (33.3)
*Klebsiella pneumoniae*	1	1	2	0	1	1	0	1	1	1	9 (6.8)
*Klebsiella* sp.	0	4	1	1	8	0	3	0	0	4	21 (15.9)
*Proteus vulgaris*	0	0	0	0	2	0	0	0	0	0	2 (1.52)
*Pseudomonas* sp.	0	1	0	0	0	0	0	0	2	0	3 (2.3)
*Staphylococcus aureus*	0	3	1	0	1	0	0	5	2	3	15 (11.4)
*Staphylococcus epidermidis*	0	1	4	0	2	0	0	1	0	0	8 (6.1)
Total (%)	3 (4.0)	20 (30.0)	11 (16.0)	3 (4.0)	30 (45.0)	6 (9.0)	11 (17.0)	19 (29.0)	8 (12.0)	21 (31.0)	132

ATS: Accra Team Station; AB: Agbogbloshie; UNM: University of Ghana Night Market; SH: Shiashi; KB: Korle-Bu.

## Data Availability

The datasets used to support the findings of this study are available on reasonable request.
